# Augmented muscle deoxygenation during repeated sprint exercise with post‐exercise blood flow restriction

**DOI:** 10.14814/phy2.15294

**Published:** 2022-05-19

**Authors:** Koki Ienaga, Keiichi Yamaguchi, Naoki Ota, Kazushige Goto

**Affiliations:** ^1^ Graduate School of Sport and Health Science Ritsumeikan University Kusatsu Shiga Japan

## Abstract

Blood flow restriction (BFR) during low‐intensity exercise has been known to be a potent procedure to alter metabolic and oxygen environments in working muscles. Moreover, the use of BFR during inter‐set rest periods of repeated sprint exercise has been recently suggested to be a potent procedure for improving training adaptations. The present study was designed to determine the effect of repeated sprint exercise with post‐exercise BFR (BFR during rest periods between sprints) on muscle oxygenation in working muscles. Eleven healthy males performed two different conditions on different days: either repeated sprint exercise with BFR during rest periods between sets (BFR condition) or without BFR (CON condition). A repeated sprint exercise consisted of three sets of 3 × 6‐s maximal sprints (pedaling) with 24s rest periods between sprints and 5 min rest periods between sets. In BFR condition, two min of BFR (100–120 mmHg) for both legs was conducted between sets. During the exercise, power output and arterial oxygen saturation (SpO_2_) were evaluated. Muscle oxygenation for the vastus lateralis muscle, exercise‐induced changes in muscle blood flow, and muscle oxygen consumption were measured. During BFR between sets, BFR condition presented significantly higher deoxygenated hemoglobin + myoglobin (*p* < 0.01) and lower tissue saturation index (*p* < 0.01) than those in CON condition. However, exercise‐induced blood lactate elevation and reduction of blood pH did not differ significantly between the conditions. Furthermore, power output throughout nine sprints did not differ significantly between the two conditions. In conclusion, repeated sprint exercise with post‐exercise BFR augmented muscle deoxygenation and local hypoxia, without interfering power output.

## 1 INTRODUCTION

A number of teams and racket sports (e.g., soccer, basketball, and hockey) require the ability to perform repeated maximal sprints (<10 s) with a short recovery (<30 s). This ability is called as repeated‐sprint ability (RSA) (Bishop et al., 2011; Girard et al., 2011). To improve RSA, repeated sprint‐exercise (RSE) is recommended. Several previous studies reported that RSE increased maximal oxygen uptake ($\dot{V}$ O_2peak_) and total work during the identical period of exercise, and improved muscle buffer capacity (Edge et al., 2005, 2006). Especially, since muscle buffer capacity plays a role in maintaining muscle pH during high‐intensity exercise, the improved capacity leads to augmented RSA (Edge, Bishop, & Goodman, 2006). Accumulation of muscle metabolites such as hydrogen ion (H^+^) and inorganic phosphate (Pi) in working muscles during a single session of repeated sprint exercise would be a physiological stimulus for improving muscle buffer capacity (Bishop et al., 2011; Weston et al., 1996). Although metabolites accumulation is closely associated with the exercise intensity and duration utilized, further increases in exercise intensity and duration may not be practical due to the elevated risk of overreaching and/or overtraining. In contrast, an alternative strategy which promotes metabolites accumulation is blood flow restriction (BFR) around working muscles (Mitchell et al., 2019; Taylor et al., 2016). Repeated sprint exercise with BFR may be an efficient procedure for the accumulation of muscle metabolites.

Several studies reported the effect of BFR during low‐intensity resistance exercise (Abe et al., 2005; Takarada et al., 2000), cycling exercise (Hwang et al., 2020) or walking (Abe et al., 2006). However, we are aware that low‐intensity exercise is generally combined with BFR. This is because high‐intensity exercise rapidly increases muscle blood flow during working muscles (Joyner & Casey et al., 2015), and the use of BFR during high‐intensity would involve the difficulty of restriction of blood flow by tourniquet. Also, high‐intensity exercise with BFR markedly impaired power output and the number of sprints during exercise (Willis et al., 2018, 2019). Impaired power output during each training session is thought to be negative for training adaptations.

To our knowledge, only two studies focused on the combined effect of sprint interval exercise with post‐exercise BFR. Taylor et al. (2016) reported the effect of 4 weeks of sprint interval training (4–7 bouts of maximal 30 s sprints) with post‐exercise BFR, and the post‐exercise BFR enhanced $\dot{V}$ O_2max_ and HIF‐1αmRNA expression compared with the same exercise training without post‐exercise BFR. Furthermore, 4 weeks of sprint interval training (4–7 bouts of maximal 30 s sprints) with post‐exercise BFR also increased $\dot{V}$ O_2max_, but no additional effect on muscle capillarity or mitochondrial protein content was observed (Mitchell et al., 2019). BFR is likely to affect dramatically muscle oxygenation environment in addition to muscle blood flow, however, the effect of a single session of repeated sprint exercise with post‐exercise BFR on muscle metabolism in working muscles has not been evaluated so far. Since the use of near‐infrared spectroscopy (NIRS) provides information about oxygen extraction and blood volume in working muscle during repeated sprint exercise (Faiss et al., 2013; Yamaguchi et al., 2021), monitoring muscle metabolism (e.g., oxygenated and deoxygenated hemoglobin, tissue saturation) using NIRS would be beneficial to understand the transient impact of post‐exercise BFR during repeated sprint exercise.

Therefore, the purpose of the present study was to determine the effect of repeated sprint exercise with post‐exercise BFR (BFR during rest periods between sprints) on muscle oxygenation in working muscles. We hypothesized that muscle deoxygenation would be augmented, and tissue saturation index would be lower during repeated sprint exercise with post‐exercise BFR, without interfering the power output. Moreover, exercise‐induced increase in muscle blood flow would be facilitated after repeated sprint exercise with post‐exercise BFR compared with the same exercise without BFR.

## 2 METHODS

### 2.1 Subjects

Eleven healthy males participated in the present study. Their age, height, weight (mean ± standard errors [SE]) were 22.5 ± 0.3 years, 173.1 ± 61.9 cm, and 65.8 ± 6.7 kg, respectively. All subjects were informed about the experimental procedures and possible risks involved in the present study, and a written informed consent was subsequently obtained. The study was approved by the Ethics committee for Human Experiments at Ritsumeikan University, and it was conducted in accordance with the Declaration of Helsinki.

### 2.2 Experimental overview

All subjects visited the laboratory three times throughout the experiment. On the first visit, a familiarization of the exercise protocol was conducted. On the second and third visits, two main conditions (BFR condition, CON condition) were performed on different days, separated by about 1 week. The order of each condition was randomized.

A familiarization condition on the first visit consisted of warm‐up followed by repeated sprint exercise with post‐exercise BFR. The subjects performed a repeated exercise protocol (two sets of 3 × 6 s maximal sprints at 7.5% BW with 24 s rest periods between sprints) as a familiarization. During 5 min rest periods after each set, post‐exercise BFR was conducted for 2 min. The applied pressure level was 100 mmHg after the first set and 120 mmHg for the second set.

**FIGURE 1:**
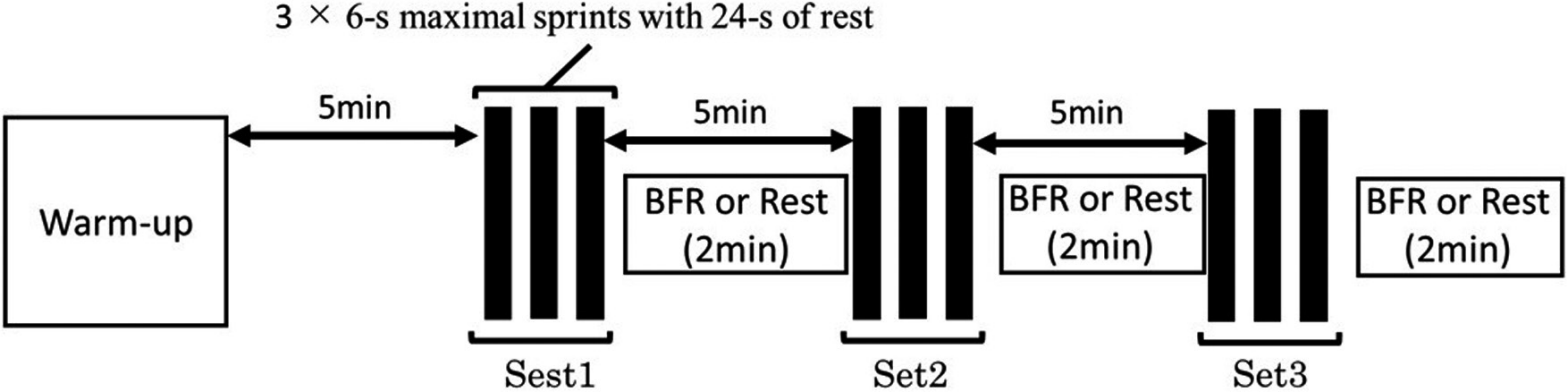
Protocol overview

Two main conditions consisted of either repeated sprint exercise with post‐exercise BFR (BFR condition) or repeated sprint exercise without post‐exercise BFR (CON condition). After completing the entire exercise session on each day, the subjects were required to remain in the supine position for 30 min. During the exercise, power output, muscle oxygenation for vastus lateralis muscle (evaluated using NIRS), arterial oxygen saturation (SpO_2_), and heart rate (HR) were evaluated. Venous blood samples were obtained before exercise, during rest periods between sets, and after completing the exercise. Additionally, muscle blood flow (mBF) and muscle blood oxygen consumption (m $\dot{V}$ O_2_) were evaluated before and after the completion of exercise. Exercise‐induced changes in these variables were compared between the two conditions (Figure 1).

### 2.3 Repeated sprint exercise

After completing baseline measurements, the subjects started prescribed warm‐up consisting of 5 min of pedaling at 90 W, followed by 4 × 3 s sprint at 50%, 60%, 70%, and 80% for maximal effort. After these submaximal sprints, a single set of 6s maximal sprints at 7.5% of BW was conducted. At 5 min after warm‐up, the subjects commenced repeated‐sprint exercises (three sets of 3 × 6 s maximal sprints with 24 s rest periods between sprints) using an electromagnetically braked cycle ergometer (Power Max XIII; Konami Corp., Tokyo, Japan). The applied load was equivalent to 7.5% BW. A 5 min of rest period was inserted after completing each set. Once the subjects completed three consecutive sprints in each set, they got off the cycle ergometer and rested on the bed with supine position. From 40 s following completing each set of sprints, post‐exercise BFR was started (Figure 1). In BFR condition, two tourniquets (SC10D; Hokanson Corp., Washington, USA) were wrapped at the proximal site of both legs. The cuff pressure achieved by the automatic pressure system (E20; Hokanson Corp., Washington, USA) was started in 40 s after the completion of each set of sprints and the applied pressure was lasted for 2 min. The pressure level was 100–120 mmHg based on the previous studies (Taylor et al., 2016; Mitchell et al., 2019). We used the minimum pressure which enabled us to provoke apparent elevation of deoxy‐Hb and reduction of TSI during post‐exercise BFR. After 2 min of cuff pressure, the tourniquets were deflated gradually, and the subjects remained in the spine position until 1 min before the start of the next sets. In CON condition, the same two tourniquets were wrapped, but the cuff pressure was not applied.

## 3 MEASUREMENTS

### 3.1 Power output during repeated sprint exercise

During repeated sprint exercises, peak and mean power output in each 6‐s sprint were evaluated.

### 3.2 Muscle oxygenation

Muscle oxygenation variables (oxygenated hemoglobin and myoglobin [oxy‐Hb + Mb], deoxygenated hemoglobin and myoglobin [deoxy‐Hb + Mb], total hemoglobin and myoglobin [total‐Hb + Mb], and tissue saturation index [TSI]) of the *vastus lateralis* of the right leg were monitored using a near‐infrared spectroscopy (NIRS) probe (Hb14; Astem Co., Ltd., Kanagawa, Japan) with an inter‐optode distance 30 mm. The NIRS probe was placed on muscle belly of *vastus lateralis* muscle (proximal 50% between the greater trochanter and border of the patella). Muscle oxygenation information (approximately 15 mm depth) was subsequently obtained.

Muscle oxygenation variables were evaluated before (during 1 min sitting on the chair before warm‐up in each condition), during each sprint (three set of 3 × 6 s sprint), and during BFR for 2 min. All signals were acquired at 10 Hz. The collected data were averaged for 6 s during each sprint set phase and for 2 min during the post‐exercise BFR phase, respectively. All data were normalized by baseline value which was collected before exercise.

### 3.3 Muscle blood flow (mBF)

The mBF was evaluated by determining the increase in the total hemoglobin and myoglobin levels during venous occlusion. Pneumatic cuff pressure (70–120 mmHg) was applied to the right leg at the proximal portion on the supine position. Subsequently, mBF was calculated using the Broatch et al. (2018) equation. The value was presented as milliliters of blood per minute, per 100 g of muscle tissue (ml min^−1^ 100 g^−1^). mBF was assessed two 20 s during venous occlusion interspersed with 1min. From the collected data, the average value was utilized. The mBF measurements were performed five times: at baseline, immediately, 10, 20, and 30 min after exercise.

### 3.4 Muscle oxygen consumption (m $\dot{V}$ O_2_)

The m $\dot{V}$ O_2_ was assessed by evaluation of the linear decrease in hemoglobin difference (Hb_diff_ = oxy – Hb + Mb – deoxy – Hb + Mb) during arterial occlusion. Pneumatic cuff pressure (250–300 mmHg) was applied to the right leg at the proximal portion on the supine position. According to the equation reported in previous study (Broach et al., 2018), m $\dot{V}$ O_2_ was calculated. Hb_diff_ was expressed in micromoles per second and conversed to milliliters O_2_ per minute per 100 g. The molecular weight of hemoglobin (64,458 g/mol), O_2_‐binding capacity of human hemoglobin (Hufner's factor = 1.39 ml O_2_/g), and a value of 1.04 Kg/l for muscle density was considered. m $\dot{V}$ O_2_ was performed three times: At baseline, 10, and 30 min after exercise. In each time point, m $\dot{V}$ O_2_ was evaluated followed by mBF, with 1 min of rest between measurements.

### 3.5 Blood variables

On the day for the main conditions, subjects visited the laboratory following an overnight fast (at least >10 h following the previous meal). A polyethylene catheter was inserted into an antecubital vein after a 15 min rest, and a baseline blood sample was obtained. Blood samples were obtained at the end of each set and 30 min after the subjects completed the exercise. All blood samples for blood gas analyses were collected using 2.5‐ml syringes with heparin treated. Blood‐gas variables including blood pH, base excess (BE), bicarbonate ion (${\text{HCO}}_{3}^{‐}$), oxygen partial pressure (pO_2_), carbon dioxide partial pressure (pCO_2_), hemoglobin (Hb), and hematocrit (Hct) levels were analyzed using an automatic blood‐gas analyzer (OPTICCA TS, Sysmex Co., Hyogo, Japan). The exercise‐induced plasma volume shift (%) was calculated using the Dill and Costill (1974) equation as follows:

$$\begin{array}{c} \Delta \text{PV}\left(\%\right)=100\times \left[\left({\text{Hb}}_{\text{pre}}/{\text{Hb}}_{\text{post}}\right)\times \left(100‐{\text{Hct}}_{\text{post}}\right)/\right.\\ \left.\;\left(100‐{\text{Hct}}_{\text{pre}}\right)‐1\right], \end{array}$$

where Hct is in % and Hb is in g/dL.

Blood lactate and glucose concentrations were measured using a lactate analyzer (Lactate Pro, Arkray Co., Kyoto, Japan) and a glucose analyzer (FreeStyle, Nipro CO., Osaka Japan), respectively.

### 3.6 SpO_2_, HR, and subjective variables

Arterial oxygen saturation (SpO_2_) was monitored every 1 s using a finger pulse oximeter on the tip of the right forefinger (Pulsox‐Me300, Teijin CO., Ltd., Tokyo Japan) throughout the exercise session. HR was continuously recorded using a wireless HR monitor (RS400, Polar Electro, Tokyo, Japan). Immediately after each set of sprints and 30 s before the end of BFR, the subjects were asked rating of perceived exertion to assess respiratory (RPE‐R) and lower limb discomfort (RPE‐L) using 10 point scales (Sumi et al., 2019).

### 3.7 Statistical analysis

Data are presented as means ± SE. A two‐way analysis of variance with repeated measures was applied using statistical software (SPSS; IBM Corp., Armonk, NY, United States) to assess the interaction and main effect (condition and time). When the ANOVA revealed a significant interaction and main effect, the Tukey–Kramer test was performed as a post hoc analysis to identify differences. *p *< 0.05 was considered to indicate statistical significance.

## 4 RESULTS

### 4.1 Power output

**FIGURE 2:**
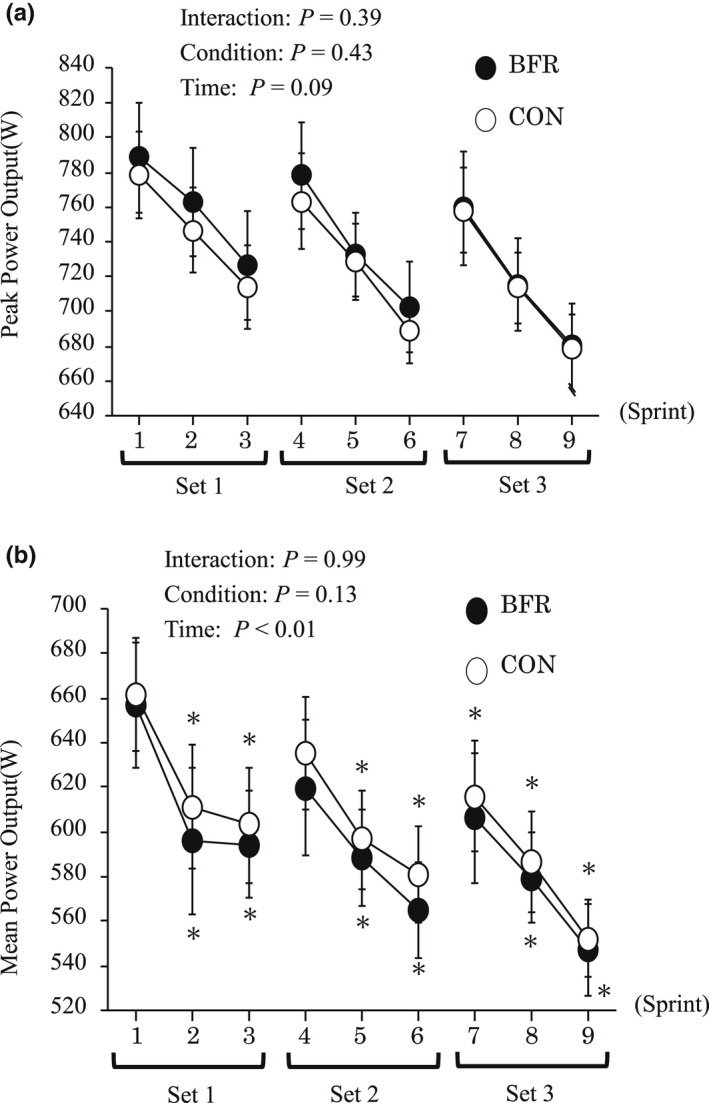
Changes in peak (a) and mean power output (b) during Sprints. Values are means ± SE. * *p *< 0.05 versus Pre.

Figure 2 presents changes in peak and mean power output during sprint exercise. There was a significant main effect of time for mean power output. However, no significant interaction or main effect of the condition were observed. Peak power output during each sprint was significantly decreased with the progress of sprints, but no significant interaction, main effects of condition or time were observed.

### 4.2 Muscle oxygenation during sprints

**FIGURE 3:**
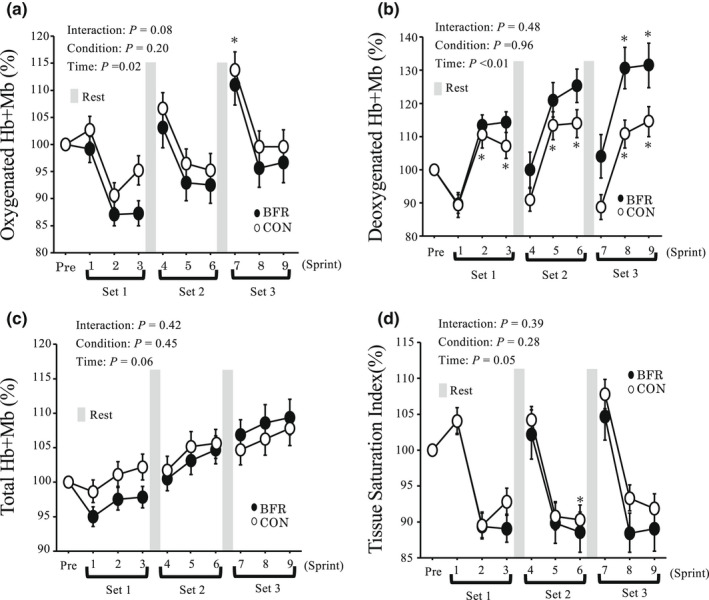
Changes Oxy‐Hb + Mb (a), Deoxy‐Hb + Mb (b), Total‐Hb + Mb (c), and TSI (d) in *vastus lateralis* muscle during Sprint. Values are means ± SE. * *p* < 0.05 versus Pre.

Figure 3 presents muscle oxygenation variables during 6 s of each sprint. The oxy‐Hb + Mb was significantly altered with sprints, whereas no significant interaction or main effect of condition were observed. The deoxy‐Hb + Mb was significantly increased from pre‐exercise value with the progress of sprints. However, no significant interaction or main effect of the condition were observed. The total‐Hb + Mb did not show significant interaction, main effects of condition or time. The TSI significantly decreased during sprints, but no significant interaction or main effect of the condition were found.

### 4.3 Muscle oxygenation during inter‐set rest periods

**FIGURE 4:**
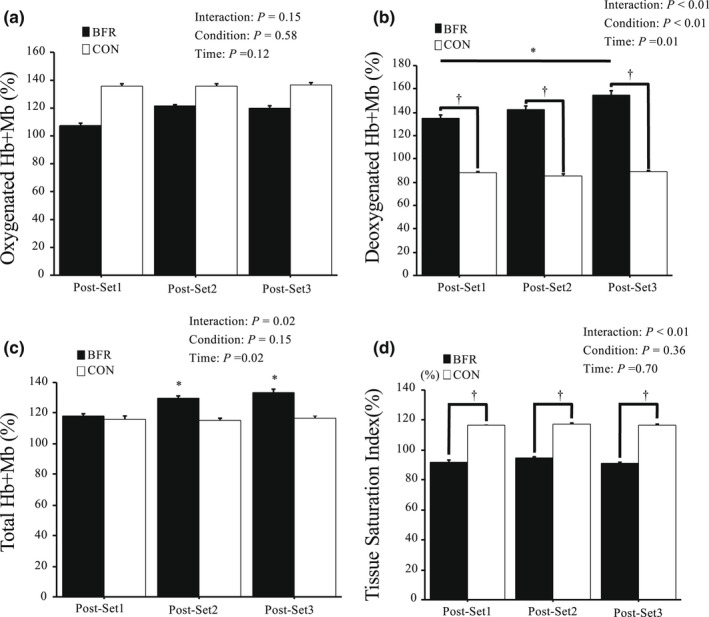
Changes Oxy‐Hb + Mb (a), Deoxy‐Hb + Mb (b), Total‐Hb + Mb (c) and TSI (d) in *vastus lateralis* during Post‐exercise BFR. Values are mean ± SE. *p < 0.05 versus Post BFR1. †*p* < 0.05 versus CON.

Figure 4 presents muscle oxygenation during inter‐set rest periods. For oxy‐Hb + Mb, no significant interaction or main effects of condition or time were observed. For deoxy‐Hb + Mb, significant interaction and the main effect of the condition were found with being significantly higher in BFR. The total‐Hb + Mb showed significant interaction and the main effect of time, with no significant main effect of condition. For TSI, a significant interaction was presented. The BFR condition presented significantly lower values than CON condition.

### 4.4 Muscle blood flow

**FIGURE 5:**
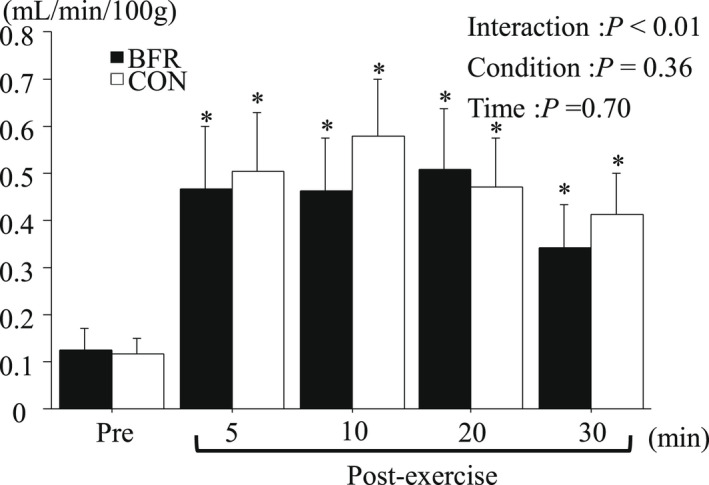
mBF to the vastus lateralis muscle at Pre and Post exercise. Values are mean ± SE. *p < 0.05 versus Pre.

Figure 5 presents mBF before and after exercise. The mBF was increased significantly after exercise in both conditions. However, no significant difference between the conditions was observed.

### 4.5 Muscle oxygen consumption

**FIGURE 6:**
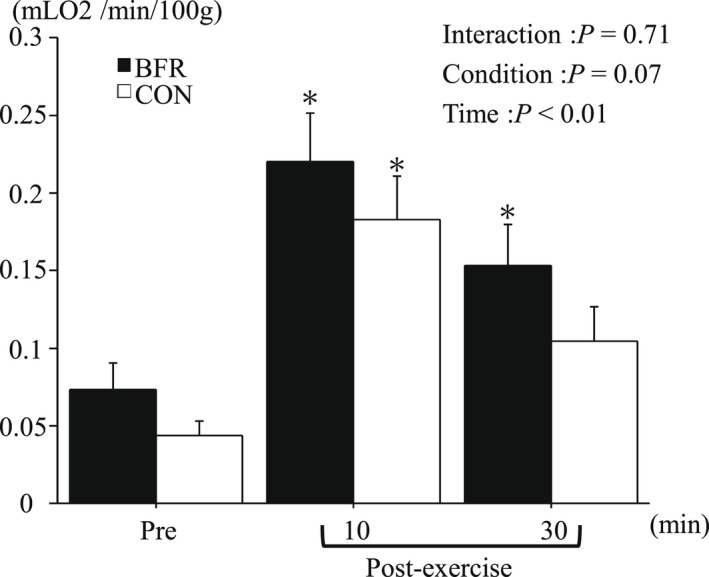
m $\dot{V}$ O_2_ to the vastus lateralis muscle at Pre and Post exercise. Values are mean ± SE. *p < 0.05 versus Pre.

Figure 6 presents m $\dot{V}$ O_2_ before and after exercise. The m $\dot{V}$ O_2_ significantly increased after exercise in both conditions. Although post‐exercise m $\dot{V}$ O_2_ tended to be higher in BFR condition, it did not reach significant difference.

### 4.6 Blood variables

Table 1 presents blood metabolites, blood gas, and plasma‐volume shift before and after exercise. Blood lactate concentrations were significantly increased in both conditions (main effect of time, *p* < 0.01). However, no significant interaction or main effect of the condition were observed. The blood glucose concentration significantly increased in both conditions (main effect of time: *p* < 0.01). However, there was no significant interaction or main effect of the condition. Blood pO_2_ was significantly increased in both conditions (main effects: *p* < 0.01), with no significant interaction or main effect of condition. Blood pCO_2_ was significantly decreased (main effect of time: *p* < 0.01), with no significant interaction or main effect of the condition. ΔPV presented significant main effect of time (*p *< 0.01). However, no significant interaction (*p* = 0.72) or main effect of condition (*p *= 0.34) was observed.

**TABLE 1:** Blood metabolites, blood gas, and plasma‐volume shift before and after exercise

	Pre	Set1	Set2	Set3	Post30min	Interaction	Condition time
Lactate (mmol/l)							
CON	1.3 ± 0.7	9.3 ± 0.6^a^	11.9 ± 0.6^a^	13.2 ± 0.7^a^	7.5 ± 0.7^a^	*p* = 0.04	*p* = 0.30
BFR	1.3 ± 0.8	8.5 ± 0.5^a^	10.9 ± 0.8^a^	12.8 ± 0.9^a^	7.8 ± 0.7^a^		*p* < 0.01
Glucose (mg/dL)							
CON	79.4 ± 2.2	83.9 ± 2.3	87 ± 2.3^a^	86.2 ± 2.7^a^	80.1 ± 2.7	*p* = 0.28	*p* = 0.11
BFR	81.2 ± 2.2	85.5 ± 2.7	89.5 ± 4.2	93.6 ± 3.0^a^	86.4 ± 2.5		*p* < 0.01
pO_2_ (kPa)							
CON	7.2 ± 0.8	10.0 ± 0.7^a^	11.5 ± 0.5^a^	12.1 ± 0.3^a^	9.0 ± 0.5^a^	*p* = 0.07	*p* = 0.63
BFR	6.7 ± 0.8	10.6 ± 0.8^a^	10.6 ± 0.8^a^	11.3 ± 0.7^a^	9.6 ± 0.5^a^		*p* < 0.01
pCO_2_ (kPa)							
CON	6.2 ± 0.4	5.7 ± 0.4	4.9 ± 0.1^a^	4.6 ± 0.1^a^	5.0 ± 0.1^a^	*p* = 0.45	*p* = 0.59
BFR	6.4 ± 0.2	5.6 ± 0.4	5.2 ± 0.4^a^	4.9 ± 0.3^a^	5.0 ± 0.1^a^		*p* < 0.01
ΔPV(%)							
CON	0.0 ± 0.0	−9.2 ± 2.2^a^	−11.8 ± 2.2^a^	−10.9 ± 2.7^a^	0.2 ± 3.3	*p* = 0.72	*p* = 0.34
BFR	0.0 ± 0.0	−7.6 ± 1.7^a^	−11.2 ± 1.6^a^	−8.8 ± 3.0^a^	3.7 ± 3.0		*p* < 0.01

Note

Values are means ± SE.

Abbreviation: PV, plasma Volume.

^a^ Significant difference versus Pre.

Blood pH was significantly decreased with exercise, with no significant difference between conditions (interaction: *p* = 0.49 main effect of time: *p* < 0.01). BE was significantly decreased with exercise (main effect of time: *p* < 0.01), but there were no interaction or main effect of the condition. The exercise‐induced reduction of ${\text{HCO}}_{3}^{‐}$ concentration did not differ significantly between the conditions (main effect of time: *p* < 0.01, Table 2).

**TABLE 2:** Acid‐Base balance before and after exercise

	Pre	Set1	Set2	Set3	Post30min	Interaction	Condition time
pH							
CON	7.36 ± 0.01	7.25 ± 0.01^a^	7.22 ± 0.01^a^	7.21 ± 0.02^a^	7.34 ± 0.01	*p* = 0.49	*p* = 0.84
BFR	7.37 ± 0.01	7.25 ± 0.02^a^	7.22 ± 0.02^a^	7.20 ± 0.02^a^	7.33 ± 0.01		*p* < 0.01
${\text{HCO}}_{3}^{‐}$(mmol/L)							
CON	27.1 ± 0.4	18.1 ± 0.3^a^	14.9 ± 0.6^a^	13.4 ± 0.6^a^	19.9 ± 0.9	*p* = 0.24	*p* = 0.73
BFR	27.2 ± 0.5	17.9 ± 0.5^a^	15.4 ± 0.6^a^	14.1 ± 0.6^a^	19.5 ± 0.8		*p* < 0.01
BE (mmol/L)							
CON	0.9 ± 0.2	−8.8 ± 0.4^a^	−11.9 ± 0.7^a^	−12.5 ± 1.3^a^	−5.4 ± 1.0^a^	*p* = 0.55	*p* = 0.97
BFR	1.3 ± 0.3	−8.8 ± 0.3^a^	−11.5 ± 0.8^a^	−13.0 ± 0.8^a^	−5.7 ± 0.8^a^		*p* < 0.01

Note

Values are means ± SE.

^a^ Significant difference versus Pre.

### 4.7 HR, SpO2, and RPE

Table 3 shows changes in HR, SpO_2_. The average HR values during each set presented significant main effect of time (*p* < 0.01). However, the average HR during exercise (BFR, 134 ± 4 bpm/min, 142 ± 5 bpm/min, 146 ± 4 bpm/min; CON, 134 ± 4 bpm/min, 141 ± 5 bpm/min, 144 ± 4 bpm/min) was not significantly different between conditions (*p* = 0.64). For average HR during post‐exercise BFR, significant main effects of time (*p* < 0.01) and interaction (*p *< 0.01) were observed. HR during post‐exercise BFR was higher in BFR condition compared with CON condition. SpO_2_ during exercise (BFR, 95.4 ± 0.7% for first set, 95.3 ± 0.8% for second set, 95.4 ± 0.8% for third set; CON, 95.3 ± 0.7% for first set, 95.1 ± 0.7% for second set, 95.7 ± 0.8% for third set) did not present significant interaction and main effects of time and condition (main effect of condition: *p* = 0.98; main effect of time: *p* = 0.71). During post‐exercise BFR, a significant main effect of time was found (*p* < 0.01). However, there was no significant interaction or main effect of condition.

**TABLE 3:** HR and SpO_2_ during Post exercise BFR

	Post‐set1	Post‐set2	Post‐set3	Interaction	Condition time
HR (beats/min)					
CON	108 ± 5	115 ± 5^b^	117 ± 4^b^	*p* < 0.01	*p* = 0.21
BFR	108 ± 5	119 ± 5^b^	124 ± 4^a^, ^b^		*p* < 0.01
SpO_2_ (%)					
CON	97.8 ± 0.3	97.2 ± 0.3	95.4 ± 0.7	*p* = 0.11	*p* = 0.74
BFR	97.5 ± 0.3	96.5 ± 0.7	96.5 ± 0.3		*p* = 0.01

Note

Values are means ± SE.

^a^ Significant difference versus CON.

^b^ Significant difference versus Post set1.

RPE‐R presented significant main effect of time (*p *< 0.01), with no significant main effect of condition (*p* = 0.84) and interaction (*p* = 0.45) during exercise and post‐exercise BFR. RPE‐L showed significant main effect of condition (*p* = 0.02) and time (*p* < 0.01), and interaction (*p* < 0.01). The BFR condition showed a significantly higher scores of RPE‐L than the CON condition during all post‐exercise BFR.

## 5 DISCUSSION

The present study investigated the effect of repeated sprint exercise with post‐exercise BFR on muscle oxygenation. Our main finding was that post‐exercise BFR caused higher deoxy‐Hb and lower TSI during inter‐set rest periods compared with the same exercise without BFR. Importantly, these metabolic modifications in working muscles did not interfere either mean or peak power output during the subsequent exercise. Furthermore, repeated sprint exercise with post‐exercise BFR did not affect exercise‐induced changes in muscle blood flow and muscle oxygen consumption, muscle oxygenation variables (e.g., oxy‐Hb + Mb, deoxy‐Hb + Mb) during sprint exercise, the exercise‐induced changes in blood variables (e.g., lactate, glucose, acid–base balance) and SpO_2_. Therefore, it appeared that BFR during inter‐set rest periods of repeated sprint exercise successfully augmented local hypoxia in working muscles without interfering power output (without impairing quality of the training session).

As we hypothesized, post‐exercise BFR altered markedly deoxy‐Hb + Mb and TSI. In BFR condition, deoxy‐Hb + Mb presented significantly higher levels during inter‐set rest periods compared with those in CON condition. Exercise with BFR has previously demonstrated elevated deoxy‐Hb + Mb compared with the same exercise without BFR (Ganesan et al., 2015; Kilgas et al., 2019). In the present study, no significant difference between the two conditions was observed for total‐Hb + Mb and oxy‐Hb + Mb. Oxy‐Hb + Mb, and deoxy‐Hb + Mb evaluated by NIRS reflect balances of oxygen supply to muscles and muscular oxygen extraction (Delorey et al., 2003; Grassi et al., 2003; Rodriguez et al., 2018). Considering that no differences in oxy‐Hb + Mb and total‐Hb + Mb were observed, elevated deoxy‐Hb + Mb appears to suggest that post‐exercise BFR augmented oxygen extraction in muscles. In addition to elevated deoxy‐Hb + Mb, TSI during inter‐set rest periods was significantly lower in BFR condition compared with those in CON condition. However, SpO_2_ did not significantly differ between the two conditions. An inconsistency between TSI and SpO_2_ reflects that lowered oxygen saturation was caused in working muscles (local hypoxia), not in blood circulation (systemic hypoxia). Previous studies reported that exercise in normobaric hypoxic conditions elicited lower SpO_2_ and TSI compared with normoxia (Willis et al., 2017, 2019; Yatsutani et al., 2020). Furthermore, the transient reduction in O_2_ partial pressure in the muscles during sprint exercise plays a key role in muscular adaptations (Hoppeler et al., 2008). Thus, it appears that lowered TSI is a preferable response for muscle adaptations.

HR during the inter‐set rest period (Post‐set 3) was significantly higher in BFR condition. Regardless of exercise, BFR itself increases HR (Iida et al., 2007; Patterson et al., 2019; Pope et al., 2013). During BFR, venous outflow from working muscles and stroke volume appears to be decreased (Patterson et al., 2019; Pope et al., 2013). These factors may cause compensatory elevation of HR to maintain the required cardiac output in BFR condition. Furthermore, post‐exercise BFR is considered to facilitate accumulations of muscle metabolites (Dankel et al., 2017; Taylor et al., 2016). Accumulated muscle metabolites stimulate muscle metaboreflex (Boushel et al., 2010), probably leading to changes in HR and cardiac output (Ichinose et al., 2010) during inter‐set. However, caution is required due to the lack of differences in exercise‐induced changes in blood pH and lactate concentrations between the conditions. Further determination of cardiac variables (e.g., cardiac output, stroke volume) will clarify the reason for elevated HR during inter‐set rest period in BFR condition.

In contrast to benefits of the BFR, the BFR during sprint exercise has previously shown to promote fatigue and aggravate power output decrement during the exercise (Willis et al., 2018; Willis, Borrani, et al., 2019; Willis, Peyrard, et al., 2019). However, it was notable that either peak or mean power output during repeated sprint exercise did not differ significantly between the two conditions. This would be meaningful because the power output during a single session of repeated sprint exercise reflects “the quality of the training”. Moreover, the power output during the training session was closely associated with increase in power output following three weeks of the training period (Ikutomo et al., 2018). Repeated sprint exercise (<6s) mainly utilizes ATP/phosphocreatine for energy substrate, and power output decrement is related to the recovery of phosphocreatine between sprints (Girard et al., 2011). Therefore, it appears that post‐exercise BFR in the present study did not delay recovery of phosphocreatine stores. Accumulated muscle metabolites (e.g., hydrogen ion, inorganic phosphate) in working muscles also limit power output during repeated sprint exercise (Girard et al., 2011). BFR augments metabolites accumulations in working muscles, but a partial duration (2 min) of BFR during 5 min of inter‐set periods did not impair significantly power output during the exercise.

Exercise‐induced change in mBF and m $\dot{V}$ O_2_ did not differ significantly between the two conditions. We initially hypothesized that BFR condition would present greater exercise‐induced elevation of mBF and m $\dot{V}$ O_2_ compared with CON condition because post‐exercise blood flow was elevated when combining the BFR during exercise (Christiansen et al., 2019; Dankel et al., 2017). Also, Broach et al. (2018) reported that 10 × 6‐s maximal sprint increased mBF during early phase of the whole exercise session. Although high‐intensity exercise drastically increases blood flow around working muscle (Joyner & Casey, 2015), it is possible that there was a ceiling effect of exercise‐induced mBF elevation, with being independent of the utilization of BFR. m $\dot{V}$ O_2_ is closely related to mBF (Joyner & Casey, 2015). In the present study, since no difference of mBF between the two conditions was observed, the lack of difference in m $\dot{V}$ O_2_ would not be surprising.

Some limitations exist in the present study. First, an optimal level of cuff pressure for post‐exercise BFR still remains unclear, although appropriate guideline of cuff pressure during exercise was proposed (Patterson et al., 2019). In addition, we did not measure blood pressure and endocrine response. Exercise with BFR elevated growth hormone and cortisol concentrations (Abe et al., 2006; Takano et al., 2005). Moreover, systolic blood pressure and mean arterial pressure were elevated immediately after exercise with BFR (Silva et al., 2019). In future study, determinations of cardiovascular response to repeated sprint exercise with post‐exercise BFR would be valuable.

In conclusion, repeated sprint exercise with post‐exercise BFR augmented deoxygenation and lowered muscle oxygen saturation in working muscles. However, repeated sprint exercise with post‐exercise BFR did not affect power output and exercise‐induced changes in SpO_2_ and blood lactate concentration. These results suggest that repeated sprint exercise with post‐exercise BFR facilitates local hypoxia in working muscles without interfering power output during exercise.

## CONFLICT OF INTEREST

The authors did not declare any conflict of interest.

## ETHICS STATEMENT

The study was approved by the Ethics committee for Human Experiments at Ritsumeikan University, and it was conducted in accordance with the Declaration of Helsinki.

## AUTHORS' CONTRIBUTION

Koki Ienaga, and Kazushige Goto were part of the conception, protocol design. Koki Ienaga, Keiichi Yamaguchi, Naoki Ota conducted the experiments. Koki Ienaga was responsible for data analyses. Koki Ienaga, and Kazushige Goto wrote the manuscript.
